# Supplementary material for: Sir2 phosphorylation through cAMP-PKA and CK2 signaling inhibits the lifespan extension activity of Sir2 in yeast
Source: eLife. 2015 Sep 2;4:e09709. doi: 10.7554/eLife.09709 (PMC4586308; doi:10.7554/eLife.09709)
Supplement: Figure 3—source data 1. — DOI: http://dx.doi.org/10.7554/eLife.09709.012 [file elife09709s001.docx]

| NO. | Mutated Gene (Systematic name) | Relative growth  (0.8 M NaCl/YPD) | NO. | Mutated Gene (Systematic name) | Relative growth  (0.8 M NaCl/YPD) |
| --- | --- | --- | --- | --- | --- |
| 1 | PSK1 (YAL017W) | 0.77 | 62 | PTK1 (YKL198C) | 0.54 |
| 2 | KIN3 (YAR018C) | 0.73 | 63 | VHS1 (YDR247W) | 1.13 |
| 3 | KNS1 (YLL019C) | 0.60 | 64 | CKA1 (YIL035C) | 0.55 |
| 4 | KIN2 (YLR096W) | 0.62 | 65 | PRK1 (YIL095W) | 0.56 |
| 5 | HOG1 (YLR113W) | 0.20 | 66 | PKP1 (YIL042C) | 0.93 |
| 6 | SKY1 (YMR216C) | 0.55 | 67 | DAK2 (YFL053W) | 0.57 |
| 7 | TDA1 (YMR291W) | 0.36 | 68 | CMK1 (YFR014C) | 0.93 |
| 8 | CLA4 (YNL298W) | 0.30 | 69 | PKH2 (YOL100W) | 1.01 |
| 9 | CKA2 (YOR061W) | 0.98 | 70 | SKM1 (YOL113W) | 1.00 |
| 10 | MEK1 (YOR351C) | 0.62 | 71 | YGK3 (YOL128C) | 0.41 |
| 11 | PSK2 (YOL045W) | 0.30 | 72 | CMK2 (YOL016C) | 0.37 |
| 12 | TPK2 (YPL203W) | 0.80 | 73 | DAK1 (YML070W) | 0.46 |
| 13 | Unknown (YPL150W) | 0.96 | 74 | SMK1 (YPR054W) | 0.70 |
| 14 | FRK1 (YPL141C) | 0.91 | 75 | IME2 (YJL106W) | 0.31 |
| 15 | MKK2 (YPL140C) | 0.72 | 76 | BCK1 (YJL095W) | 0.52 |
| 16 | Unknown (YPL109C) | 1.05 | 77 | IKS1 (YJL057C) | 0.35 |
| 17 | KIN1 (YDR122W) | 0.66 | 78 | BUB1 (YGR188C) | 0.33 |
| 18 | GUT1 (YHL032C) | 1.02 | 79 | THR1 (YHR025W) | 0.43 |
| 19 | STE20 (YHL007C) | Very slow growth | 80 | PRR2 (YDL214C) | 0.71 |
| 20 | SLT2 (YHR030C) | Very slow growth | 81 | CHK1 (YBR274W) | 0.44 |
| 21 | IRE1 (YHR079C) | 0.90 | 82 | PTK2 (YJR059W) | 0.71 |
| 22 | KSP1 (YHR082C) | 0.61 | 83 | DUN1 (YDL101C) | 0.43 |
| 23 | YCK1 (YHR135C) | 0.62 | 84 | STE7 (YDL159W) | 0.39 |
| 24 | KCC4 (YCL024W) | 0.63 | 85 | ARK1 (YNL020C) | 0.22 |
| 25 | SAT4 (YCR008W) | 0.63 | 86 | URK1 (YNR012W) | 0.37 |
| 26 | CKI1 (YLR133W) | 0.33 | 87 | SSK2 (YNR031C) | 0.48 |
| 27 | MET14 (YKL001C) | 0.39 | 88 | FPK1 (YNR047W) | 1.30 |
| 28 | ELM1 (YKL048C) | 0.32 | 89 | YPK3 (YBR028C) | 0.66 |
| 29 | HSL1 (YKL101W) | 0.39 | 90 | AKL1 (YBR059C) | 0.42 |
| 30 | PRR1 (YKL116C) | 0.29 | 91 | KSS1 (YGR040W) | 1.02 |
| 31 | YPK1 (YKL126W) | 0.56 | 92 | DBF2 (YGR092W) | 1.20 |
| 32 | KDX1 (YKL161C) | 1.02 | 93 | YAK1 (YJL141C) | 1.07 |
| 33 | TPK3 (YKL166C) | 0.75 | 94 | HAL5 (YJL165C) | 0.00 |
| 34 | KKQ8 (YKL168C) | 0.44 | 95 | CTK1 (YKL139W) | 0.14 |
| 35 | NNK1 (YKL171W) | 0.61 | 96 | SSK22 (YCR073C) | 0.01 |
| 36 | FMP48 (YGR052W) | 1.01 | 97 | RIM11 (YMR139W) | 0.47 |
| 37 | LCB4 (YOR171C) | 0.36 | 98 | HRK1 (YOR267C) | 0.42 |
| 38 | MKK1 (YOR231W) | 0.48 | 99 | FAB1 (YFR019W) | 0.38 |
| 39 | KIN4 (YOR233W) | 0.46 | 100 | PBS2 (YJL128C) | 0.21 |
| 40 | SWE1 (YJL187C) | 0.89 | 101 | YPK2 (YMR104C) | 0.38 |
| 41 | HAL5 (YJL165C) | 0.76 | 102 | RTK1 (YDL025C) | 0.62 |
| 42 | TPK1 (YJL164C) | 0.75 | 103 | MRK1 (YDL079C) | 0.40 |
| 43 | STE11 (YLR362W) | 0.62 | 104 | PKH3 (YDR466W) | 0.50 |
| 44 | VIP1 (YLR410W) | 0.23 | 105 | PKH1 (YDR490C) | 0.39 |
| 45 | RCK2 (YLR248W) | 0.42 | 106 | GIN4 (YDR507C) | 0.51 |
| 46 | MCP2 (YLR253W) | 0.41 | 107 | RCK1 (YGL158W) | 0.59 |
| 47 | LCB5 (YLR260W) | 0.33 | 108 | TOS3 (YGL179C) | 0.43 |
| 48 | EKI1 (YDR147W) | 0.33 | 109 | YCK3 (YER123W) | 0.36 |
| 49 | ADK1 (YDR226W) | 1.12 | 110 | SAK1 (YER129W) | 0.34 |
| 50 | SSN3 (YPL042C) | Very slow growth | 111 | TOR1 (YJR066W) | 0.78 |
| 51 | ISR1 (YPR106W) | 0.71 | 112 | SNF1 (YDR477W) | 0.44 |
| 52 | DBF20 (YPR111W) | 0.60 | 113 | SPS1 (YDR523C) | 0.53 |
| 53 | KIN82 (YCR091W) | 0.64 | 114 | RIM15 (YFL033C) | 0.27 |
| 54 | ALK2 (YBL009W) | 0.42 | 115 | VPS34 (YLR240W) | 0.01 |
| 55 | FUS3 (YBL016W) | 0.49 | 116 | PHO85 (YPL031C) | 0.01 |
| 56 | TEL1 (YBL088C) | 0.40 | 117 | GCN2 (YDR283C) | 0.35 |
| 57 | ALK1 (YGL021W) | 0.53 | 118 | PRO1 (YDR300C) | 0.56 |
| 58 | PKP2 (YGL059W) | 0.53 | 119 | BUD32 (YGR262C) | 0.68 |
| 59 | SCY1 (YGL083W) | 0.48 | 120 | VPS15 (YBR097W) | 0.14 |
| 60 | NPR1 (YNL183C) | 1.23 | 121 | MCK1 (YNL307C) | 0.44 |
| 61 | YCK2 (YNL154C) | 1.08 | WT | BY4741 (Parent strain) | 0.75 |
