# Supplementary material for: Sir2 phosphorylation through cAMP-PKA and CK2 signaling inhibits the lifespan extension activity of Sir2 in yeast
Source: eLife. 2015 Sep 2;4:e09709. doi: 10.7554/eLife.09709 (PMC4586308; doi:10.7554/eLife.09709)
Supplement: Supplementary file 1. — Strains used in the study. DOI: http://dx.doi.org/10.7554/eLife.09709.016 [file elife09709s002.pdf]

Supplementary Table 1. Strains used in the study.

Strain	Genotype	Reference
BY4741 (Wild type)	*MATa his3Δ1 leu2Δ0 met15Δ0 ura3Δ0*	Lab. collection
sir2Δ	*sir2Δ::tc*	This work
fob1Δ	*fob1Δ::tc*	This work
sir2Δ fob1Δ	*sir2Δ::tc fob1Δ::tc*	This work
sir2Δ hmlΔ [*URA3*]	*sir2Δ::tc hmlΔ::tc-URA3-tc*	This work
sir2Δ hmlΔ	*sir2Δ::tc hmlΔ::tc*	This work
WT [*PMA1-FLAG*]	*pma1::PMA1-FLAG-URA3*	This work
sir2Δ [*PMA1-FLAG*]	*sir2Δ::tc pma1::PMA1-FLAG-URA3*	This work
WT [*SIR2-URA3*]	*ura3Δ0::SIR2-URA3*	This work
pde2Δ [*SIR2-URA3*]	*pde2Δ::tc ura3Δ0::SIR2-URA3*	This work
WT [*URA3*]	*ura3Δ0::URA3*	This work
sir2Δ [*URA3*]	*sir2Δ ura3Δ0::URA3*	This work
sir2Δ [*SIR2*]	*sir2Δ::SIR2-URA3*	This work
sir2Δ [*sir2-H364Y*]	*sir2Δ::sir2-H364Y-URA3*	This work
WT [*Sir2-S473E*]	*sir2Δ::sir2-S473E-URA3*	This work
WT [*Sir2-S473A*]	*sir2Δ::sir2-S473A-URA3*	This work
pde2Δ [*Sir2-S473A*]	*pde2Δ::tc sir2Δ::sir2-S473A-URA3*	This work
sas2Δ	*sas2Δ::tc*	This work
sir2Δ sas2Δ	*sir2Δ*::tc *sas2Δ::tc*	This work
sir3Δ	*sir3Δ::tc*	This work
sir2Δ sir3Δ	*sir2Δ*::tc *sir3Δ::tc*	This work
sir4Δ	*sir4Δ::tc*	This work
sir2Δ sir4Δ	*sir2Δ*::tc *sir4Δ::tc*	This work
pde2Δ	*pde2Δ::tc*	This work
sir2Δ pde2Δ	*sir2Δ*::tc *pde2Δ::tc*	This work
pde2Δ tpk1Δ	*pde2Δ::tc tpk1Δ::tc*	This work
pde2Δ tpk1Δ tpk2Δ	*pde2Δ::tc tpk1Δ::tc tpk2Δ::tc*	This work
pde2Δ tpk1Δ tpk2Δ tpk3Δ	*pde2Δ::tc tpk1Δ::tc tpk2Δ::tc tpk3Δ::tc*	This work
sir2Δ pde2Δ hst1Δ	*sir2Δ*::tc *pde2Δ::tc hst1Δ::tc*	This work
sir2Δ pde2Δ hst2Δ	*sir2Δ*::tc *pde2Δ::tc hst2Δ::tc*	This work
sir2Δ pde2Δ hst3Δ	*sir2Δ*::tc *pde2Δ::tc hst3Δ::tc*	This work
sir2Δ pde2Δ hst4Δ	*sir2Δ*::tc *pde2Δ::tc hst4Δ::tc*	This work
cka2Δ	*cka2Δ::tc*	This work
sir2Δ cka2Δ	*sir2Δ*::tc *cka2Δ::tc*	This work
pde2Δ cka2Δ	*pde2Δ::tc cka2Δ::tc*	This work
cka2Δ [*Sir2-S473A*]	*cka2Δ::tc sir2Δ::sir2-S473A-URA3*	This work
cka2Δ [*Sir2-S473E*]	*cka2Δ::tc sir2Δ::sir2-S473E-URA3*	This work
WT [*SIR2-13 Myc*]	*sir2::SIR2-13 Myc-URA3*	This work
pde2Δ [*SIR2-13 Myc*]	*pde2Δ::tc sir2::SIR2-13 Myc-URA3*	This work
pde2Δ cka2Δ [*SIR2-13 Myc*]	*pde2Δ::tc cka2Δ::tc sir2::SIR2-13 Myc-URA3*	This work
WT [*CKA2-FLAG*]	*cka2::CKA2-FLAG-URA3*	This work
sir2Δ [*CKA2-FLAG*]	*sir2Δ*::tc *cka2::CKA2-FLAG-URA3*	This work
pde2Δ [*CKA2-FLAG*]	*pde2Δ::tc cka2::CKA2-FLAG-URA3*	This work
pde2Δ tpk1Δ tpk2Δ tpk3Δ [*CKA2-FLAG*]	*pde2Δ::tc tpk1Δ::tc tpk2Δ::tc tpk3Δ::tc cka2::CKA2-FLAG-URA3*	This work
kss1Δ	*kss1Δ::tc*	This work
sir2Δ kss1Δ	*sir2Δ*::tc *kss1Δ::tc*	This work
pde2Δ kss1Δ	*pde2Δ*::tc *kss1Δ::tc*	This work
dbf2Δ	*dbf2Δ::tc*	This work
sir2Δ dbf2Δ	*sir2Δ*::tc *dbf2Δ::tc*	This work
pde2Δ dbf2Δ	*pde2Δ*::tc *dbf2Δ::tc*	This work
BY4741	*MATa his3Δ1 leu2Δ0 met15Δ0 ura3Δ0*	Kaeberlein lab.
BY4741 *sir2Δ*	*MATa his3Δ1 leu2Δ0 met15Δ0 ura3Δ0 sir2Δ::URA3*	Kaeberlein lab.
BY4742	*MATα his3Δ1 leu2Δ0 lys2Δ0 ura3Δ0*	Kaeberlein lab.
BY4742 *sir2Δ*	*MATα his3Δ1 leu2Δ0 lys2Δ0 ura3Δ0 sir2Δ::LEU2*	Kaeberlein lab.
W303	*MATa leu2-3,112 trp1-1 can1-100 ura3-1 ade2-1 his3-11,15*	Kaeberlein lab.
W303 *sir2Δ*	*MATa leu2-3,112 trp1-1 can1-100 ura3-1 ade2-1 his3-11,15 sir2Δ::ADE2*	Kaeberlein lab.
PSY316	*MATa ura3–52 leu2-3,112 his3- 200 ade2–101 lys2–801*	Kaeberlein lab.
PSY316 *sir2Δ*	*MATa ura3–52 leu2-3,112 his3- 200 ade2–101 lys2–801 sir2Δ::ADE2*	Kaeberlein lab.
10560-2B (Parent strain)	*MATa ura3-52 leu3Δ::hisG his3Δ::hisG*	Lab. collection
sir2Δ	10560-2B *sir2Δ::tc*	This work
ra2Δ	10560-2B *ras21Δ::tc*	This work
sir2Δ ras2Δ	10560-2B *sir2Δ::tc ras2Δ::tc*	This work
gpr1Δ	10560-2B *gpr1Δ::tc*	This work
sir2Δ gpr1Δ	10560-2B *sir2Δ::tc fpr1Δ::tc*	This work
