# Supplementary material for: Sir2 phosphorylation through cAMP-PKA and CK2 signaling inhibits the lifespan extension activity of Sir2 in yeast
Source: eLife. 2015 Sep 2;4:e09709. doi: 10.7554/eLife.09709 (PMC4586308; doi:10.7554/eLife.09709)
Supplement: Supplementary file 2. — Primers used in the study. DOI: http://dx.doi.org/10.7554/eLife.09709.017 [file elife09709s003.pdf]

Supplementary Table 2. Primers used in the study.

Primer	Sequence (5' to 3')	Description
ACT1-F	AGCCTTCTACGTTTCCATCCA	qRT-PCR for *ACT1* (390)
ACT1-R	TAACACCATCACCGGAATCCA	qRT-PCR for *ACT1* (478)
PMA1-F	CTTCAGCATCTTCTGTTTCAGC	qRT-PCR for *PMA1* (32)
PMA1-R	CGTCAGAAGATTCAGATGCAG	qRT-PCR for *PMA1* (98)
SIR2-F	GTAGTGTCAATGTCCAATGACG	qRT-PCR for *SIR2* (1289)
SIR2-R	TCGAGCATTGAGAGACTCTC	qRT-PCR for *SIR2* (1417)
RPL1A-qPCR-F	GACTTGTACGGTAAGGTCACTG	qRT-PCR for *RPL1A* (427)
RPL1A-qPCR-R	CGTCTTCTTCCATTTCAACG	qRT-PCR for *RPL1A* (532)
RPL1B-qPCR-F	CGATGACTTGTACGGTAAGG	qRT-PCR for *RPL1B* (442)
RPL1B-qPCR-R	CGTCTTCTTCCATTTCAACG	qRT-PCR for *RPL1B* (532)
RPL3-qPCR-F	GGTTGTATCCCAGGTAACAG	qRT-PCR for *RPL3* (976)
RPL3-qPCR-R	GCTGACTTCTTCCAAAGCC	qRT-PCR for *RPL3* (1065)
RPL5-qPCR-F	CCTAGCTGACGACATTGATG	qRT-PCR for *RPL5* (678)
RPL5-qPCR-R	GTCAGCTCTGATAGCTTCGTG	qRT-PCR for *RPL5* (750)
RPL4A-qPCR-F	CACTTTGCCATCCCATATCATC	qRT-PCR for *RPL4A* (822)
RPL4A-qPCR-R	CGTGAGTACGCTTTTGAGTAGC	qRT-PCR for *RPL4A* (934)
RPL8B-qPCR-F	CCACCATTGACGCTAACTTCGC	qRT-PCR for *RPL8B* (647)
RPL8B-qPCR-R	GTCTTAGCTCTCTTGTCCATC	qRT-PCR for *RPL8B* (755)
RPL32-qPCR-F	CCATGCACACCAAGACTTAC	qRT-PCR for *RPL32* (276)
RPL32-qPCR-R	GGTGACCTTGATACCCAAAGCC	qRT-PCR for *RPL32* (360)
RPL37A-qPCR-F	GTGGTCGTCGTTCTTTCCATG	qRT-PCR for *RPL37A* (424)
RPL37A-qPCR-R	GTGTCTTCTCTTAGCCTTGGC	qRT-PCR for *RPL37A* (530)
RPL42B-qPCR-F	CAATCTGGTTTCGGTGGTC	qRT-PCR for *RPL42B* (580)
RPL42B-qPCR-R	GTGCTTACATCTCTTCAAGG	qRT-PCR for *RPL42B* (711)
RPL25-qPCR-F	CGAAGTTGACGTATTGAAGG	qRT-PCR for *RPL25* (723)
RPL25-qPCR-R	CTGTTAGCAATGTCCAAAGC	qRT-PCR for *RPL25* (827)
RPS0A-qPCR-F	CCCTGTCATTGCTCTAACTG	qRT-PCR for *RPS0A* (423)
RPS0A-qPCR-R	CAATGGAGTGCTTACCTCTG	qRT-PCR for *RPS0A* (492)
RPS0B-qPCR-F	GGTCCATCATGCCAGATTTG	qRT-PCR for *RPS0B* (943)
RPS0B-qPCR-R	CTTCAGCTTGACCTTCAGTGAC	qRT-PCR for *RPS0B* (1074)
RPS3-qPCR-F	GCTAAGAGCAGAACTGGTCC	qRT-PCR for *RPS3* (577)
RPS3-qPCR-R	CAGCTGGTCTGTAGTCCTTG	qRT-PCR for *RPS3* (685)
RPS7A-qPCR-F	GATCCAGAACCTTGACTGCTG	qRT-PCR for *RPS7A* (742)
RPS7A-qPCR-R	GGATCTTGTTACCACCAACC	qRT-PCR for *RPS7A* (849)
RPS13-qPCR-F	CTTGAAGTCCAATGGTTTGGC	qRT-PCR for *RPS13* (761)
RPS13-qPCR-R	CTGAACTTAGCGTCTTTGTCC	qRT-PCR for *RPS13* (880)
RPS20-qPCR-F	CTAAGACTTGGGAAACCTACG	qRT-PCR for *RPS20* (227)
RPS20-qPCR-R	CATCCACACCAGGTTCAATG	qRT-PCR for *RPS20* (340)
RPS25A-qPCR-F	GGTGGTTCTTTAGCTAGAATTGC	qRT-PCR for *RPS25A* (214)
RPS25A-qPCR-R	GAAGCAGTAGCTCTGGTGTAG	qRT-PCR for *RPS25A* (320)
RPS30A-qPCR-F	CTAGCTCGTGCTGGTAAAGTC	qRT-PCR for *RPS30A* (452)
RPS30A-qPCR-R	CGTTAACGAATCTTCTGGTGTAC	qRT-PCR for *RPS30A* (569)
RPS30B-qPCR-F	GTTCACGGTTCTCTAGCTCG	qRT-PCR for *RPS30B* (421)
RPS30B-qPCR-R	CGTTAACGAATCTTCTGGTG	qRT-PCR for *RPS30B* (550)
TEL-VIR-F	GTGCAAGCGTAACAAAGCCA	ChIP for *TEL-VIR* (-668)
TEL-VIR-R	AAGTAGTCCAGCCGCTTGTT	ChIP for *TEL-VIR* (-601)
HMR-F	AGCATTACGAAGATTCTCGATTCC	ChIP for *HMR* (532)
HMR-R	CGCCTACCTTCTTGAACAAGAT	ChIP for *HMR* (632)
-2 kb-F	GGAGCATCGTTCGTACCATC	ChIP for -2 kb upstream from *PMA1* promoter (-1926)
-2 kb-R	GTCTTAGCAAGGTCTTCGCC	ChIP for -2 kb upstream from *PMA1* promoter (-1841)
PMA1p-F	CTGGGATCACCCATACATCACT	ChIP for *PMA1* promoter (-900)
PMA1p-R	TGCGGTAATTATCGCCGGATCT	ChIP for *PMA1* promoter (-803)
RPL3p-F	GCACTCGCAAACTTGCTGC	ChIP for *RPL3* promoter (-353)
RPL3p-R	CCAGTGTGCGTGAGTAGAG	ChIP for *RPL3* promoter (-285)
RPL5p-F	GCTAACTTGTACATCCAGACATCC	ChIP for *RPS5* promoter (-302)
RPL5p-R	CGCATTGAAAGCCACCAG	ChIP for *RPS5* promoter (-199)
RPS0Ap-F	GGATGCGCTTCACAAGAATAG	ChIP for *RPS0A* promoter (-559)
RPS0Ap-R	CGCTCAGCGGCATAATAC	ChIP for *RPS0A* promoter (-431)
